# The Odyssey of Bioactive Compounds in Avocado (*Persea americana*) and Their Health Benefits

**DOI:** 10.3390/antiox8100426

**Published:** 2019-09-24

**Authors:** Deep Jyoti Bhuyan, Muhammad A. Alsherbiny, Saumya Perera, Mitchell Low, Amrita Basu, Okram Abemsana Devi, Mridula Saikia Barooah, Chun Guang Li, Konstantinos Papoutsis

**Affiliations:** 1 NICM Health Research Institute, Western Sydney University, Penrith, NSW 2751, Australia or m.alsherbiny@westernsydney.edu.au (M.A.A.); Saumya.P@westernsydney.edu.au (S.P.); Mitchell.Low@westernsydney.edu.au (M.L.); C.Li@westernsydney.edu.au (C.G.L.); 2 Department of Pharmacognosy, Faculty of Pharmacy, Cairo University, Cairo 11562, Egypt; 3 Research Centre for Toxic Compounds in the Environment, Masaryk University, Brno 62500, Czech; amrita@recetox.muni.cz; 4 Department of Food Science and Nutrition, College of Community Science, Assam Agricultural University, Assam 785013, India; okramabemsana@gmail.com (O.A.D.); mridulabarooah@aau.ac.in (M.S.B.); 5 School of Agriculture and Food Science, University College Dublin, Belfield, Dublin 4, Ireland; kostas.papoutsis@ucd.ie

**Keywords:** avocado, *Persea americana*, metabolites, antioxidants, anticancer, antimicrobial, anti-inflammatory, diabetes, cardiovascular diseases (CVD), bioavailability and pharmacokinetic

## Abstract

*Persea americana*, commonly known as avocado, has recently gained substantial popularity and is often marketed as a “superfood” because of its unique nutritional composition, antioxidant content, and biochemical profile. However, the term “superfood” can be vague and misleading, as it is often associated with unrealistic health claims. This review draws a comprehensive summary and assessment of research performed in the last few decades to understand the nutritional and therapeutic properties of avocado and its bioactive compounds. In particular, studies reporting the major metabolites of avocado, their antioxidant as well as bioavailability and pharmacokinetic properties, are summarized and assessed. Furthermore, the potential of avocado in novel drug discovery for the prevention and treatment of cancer, microbial, inflammatory, diabetes, and cardiovascular diseases is highlighted. This review also proposes several interesting future directions for avocado research.

## 1. Introduction

*Persea americana* (commonly known as avocado, avocado pear, or alligator pear) is native to Mexico and Central America, and a member of the flowering plant family Lauraceae [1,2]. Botanically, avocado fruit is a berry with a single large seed [3]. Mexico is the leading producer of avocados worldwide [2]. The term “superfood” refers to foods that are beneficial to human health due to their high levels of nutrients and/or bioactive phytochemicals such as antioxidants [4]. In particular, avocado has recently gained dramatic popularity [5] and is often referred to as a “superfood” because of its unique nutritional and phytochemical composition compared to other fruits. This has led to an exponential increase in avocado consumption from 2.23 pounds per capita in 2000 to 7.1 pounds per capita in 2016 in the United States [6]. However, the term “superfood” has been used ambiguously in popular media, and often marketed with misleading health claims of preventing and curing ailments. Considering their immense popularity and diverse biochemical content, avocados have also been extensively used in the food, nutraceutical, pharmaceutical, and cosmetic industries. In addition, their health-benefiting properties have been investigated in a number of preclinical and clinical studies in the last few decades. The present review article is focused on the comprehensive summary and assessment of research performed to understand the role of avocado and its bioactive compounds in the prevention and treatment of various ailments, including cancer, microbial, inflammatory, diabetes and cardiovascular diseases. The studies emphasizing the nutritional composition of avocado, its major metabolites, and their pharmacokinetic properties are also reviewed and summarized. Furthermore, this review highlights several interesting aspects for future research on avocado.

### 1.1. The Vast Array of Secondary Metabolites of Avocado and Their Biological Significance

**Figure 1:**
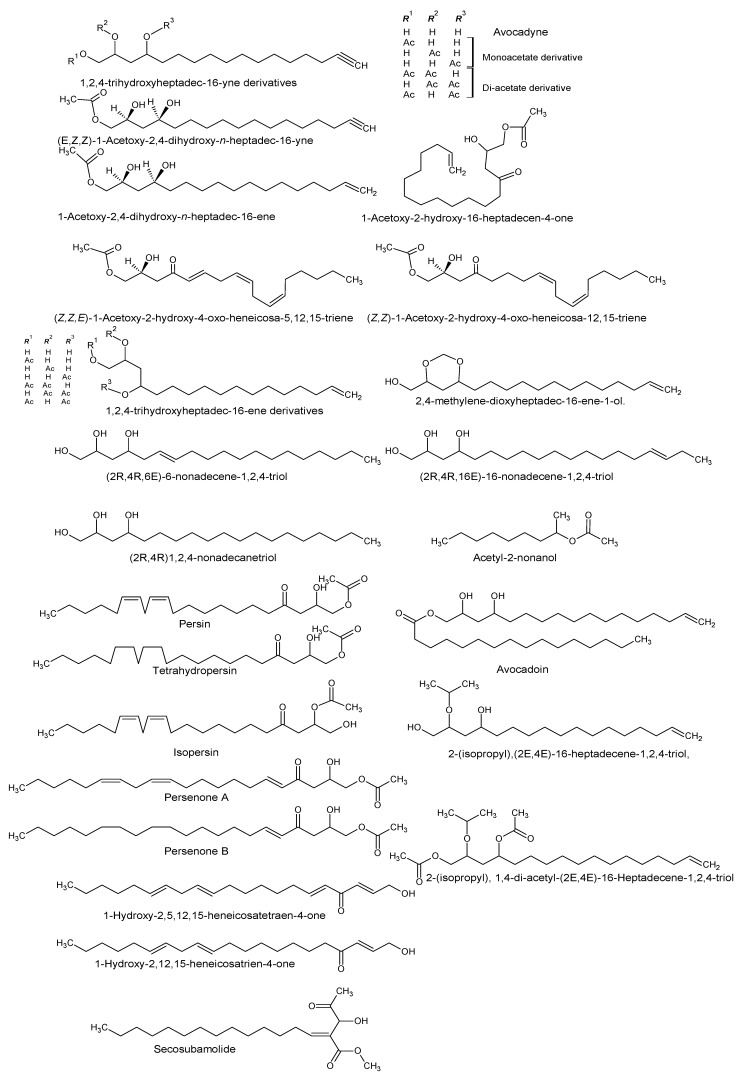
Fatty alcohols isolated from avocado.

**Figure 2:**
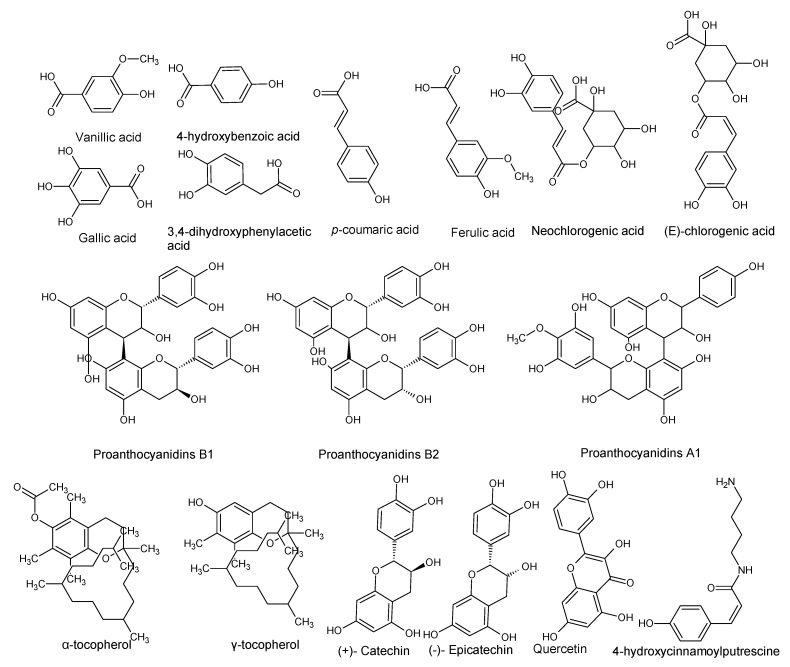
Phenolic compounds isolated from avocado.

**Figure 3:**
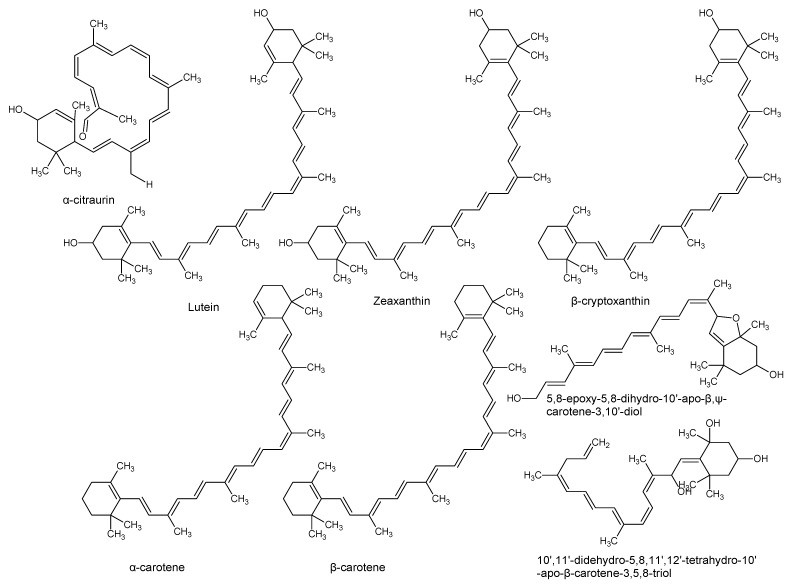
Carotenoids isolated from avocado.

**Figure 4:**
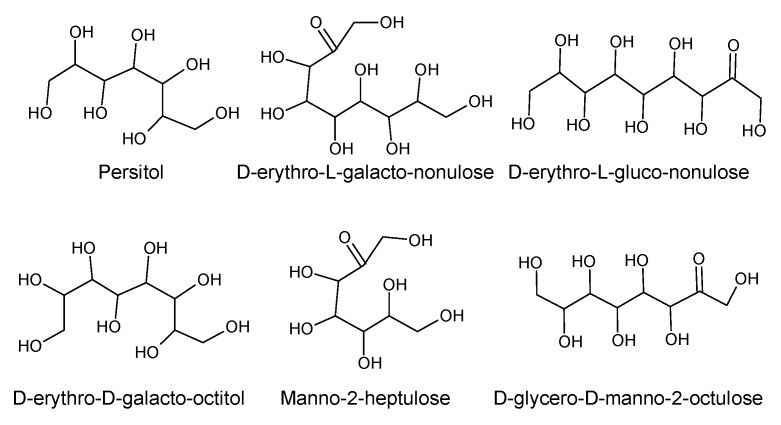
Sugars and sugar alcohol isolated from avocado.

**Figure 5:**
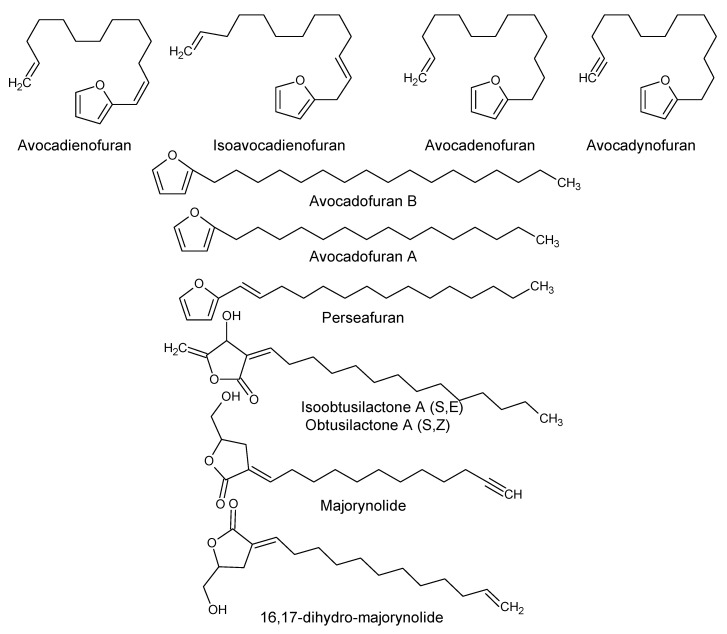
Furan and furanone derivatives isolated from avocado.

**Figure 6:**
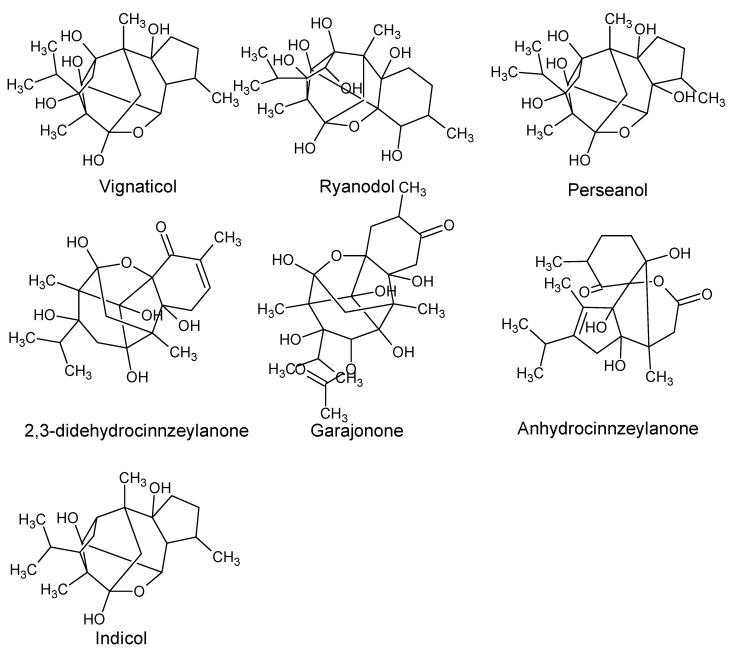
Diterpenoids isolated from avocado.

**Figure 7:**
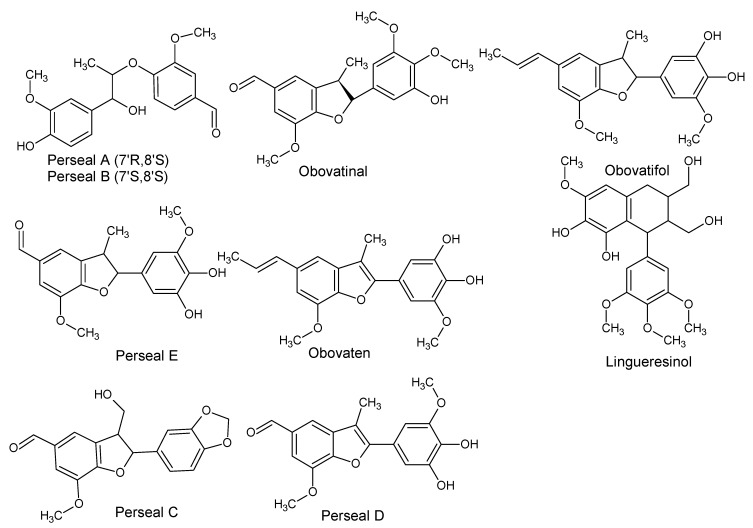
Norlignans, neolignans, and lignans isolated from avocado.

**Figure 8:**
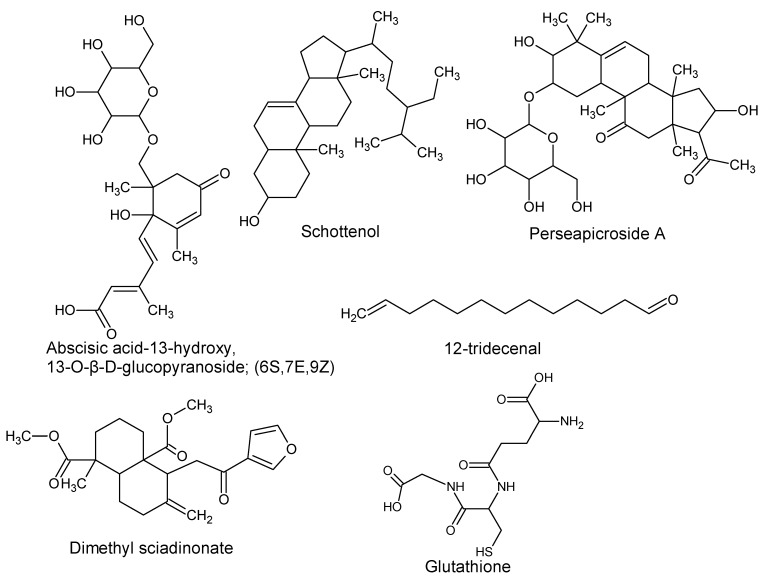
Miscellaneous compounds isolated from avocado.

Using “Avocado” and “*Persea*” as search descriptors with a focus for pharmacologically active metabolites, various avocado metabolites were retrieved from Combined Chemical Dictionary v23.1 (CCD) [7] and The Human Metabolite Database (HMDB) [8]. In addition to the *P. americana*, the search strategy also covered other *Persea* species such as *P. mexicana*, *P. indica*, *P. gratissima*, *P. obovatifolia*, and *P. borbonia* (Table 1). As per the literature, most bioactive compounds were isolated predominantly from *P. americana*. Other synonyms of *P. americana* are *P. gratissima*, *Laurus persea, P. drymifolia*, and *P. nubigena* [9]. The metabolite arsenal can be classified chemically into eight main classes, including fatty alcohols, furan derivatives, carotenoids, carbohydrate, diterpenoids, lignan derivatives, and miscellaneous compounds, as shown in Figure 1, Figure 2, Figure 3, Figure 4, Figure 5, Figure 6, Figure 7 and Figure 8, and Table 1. In brief, fatty alcohols isolated from avocado showed different degrees of unsaturation and alkyl chain length with several levels of hydroxylation and subsequent acetylation (Figure 1). These fatty alcohols have been reported to exhibit antiviral, cytotoxic, antifungal, trypanocidal, and antioxidant activity [10,11,12,13,14,15,16,17,18,19,20,21]. Phenolic compounds (Figure 2, and Table 1) of different chemical classes from simple organic acids such as gallic acid to larger flavonoids, anthocyanidins, and tocopherols were isolated from *Persea* species with significant antioxidant, neuroprotective and cardioprotective activities [22,23,24,25,26,27,28]. The antioxidant properties of avocado were also ascribed to their carotenoid content in many studies [24,28,29,30] (Figure 3). Moreover, sugar alcohol and ketoses with variable carbon chain length were isolated from avocado (Figure 4). Notable insecticidal, cytotoxic, and antifungal activities were also reported for the furan and furanone derivatives isolated from *Persea* species [18,31,32,33,34,35,36,37] (Figure 5), where the saturation of the furan ring was detrimental for the insecticidal activity [38]. The insecticidal activity of the furan derivatives was augmented by the diterpenoids compounds [39,40,41,42,43], especially in *P. indica* (Figure 6). Overall, avocado contains a vast array of secondary metabolites of different chemical classes which may attribute to its diverse biological activities.

### 1.2. Nutritional Composition of P. americana

Avocados have been recognized for their high nutritional value and therapeutic importance for centuries. The nutritional composition of avocado is shown in Table 2 according to the United States Department of Agriculture (USDA) [61]. A whole avocado is reported to contain 140 to 228 kcal (~585–1000 kJ) of energy depending on the size and variety [62]. The variety, grade of ripening, climate, the composition of the soil, and fertilizers are the major factors that largely influence the nutritional profiles of avocados [63].

Fiber constitutes most of its carbohydrate content (~9 g of fiber and 12 g of carbohydrate per avocado) (Table 2) and can reach up to 13.5 g in larger avocados. Higher quantities of insoluble and soluble fibers (70% and 30%, respectively) are found in the pulp [3]. A single serving can provide about 2 g protein and 2 g of fiber with a glycemic index of 1 ± 1 [64]. A high-fiber diet is often linked with a healthy digestive system. Moreover, it may help lower blood cholesterol levels and prevent constipation by improving bowel movement. In particular, avocados have been shown to improve the microflora of the intestines by working as a prebiotic [65]. In addition to fat, avocados are rich in protein (highest among fruits), sugars including sucrose and 7-carbon carbohydrates (d-mannoheptulose), antioxidants, pigments, tannins, and phytoestrogens [66].

Fat contributes to most of the calories in an avocado. A 1000-kJ portion of avocado contains about 25 g of fat, most of which are healthier monounsaturated fatty acids (MUFA) [64]. The lipid content in avocados is higher than in other fruits. Most lipids found in avocados are polar lipids (glycolipids and phospholipids), which play a fundamental role in various cellular processes such as the functioning of the cell membranes as second messengers [67]. These lipids are also used to make emulsions of water and lipids, and have a wide variety of applications in food, pharmaceuticals, and cosmetics industries [68]. Compared to other vegetable oils, avocado oils are high in MUFA (oleic and palmitoleic acids) and low in polyunsaturated fatty acids (linoleic acid and linolenic acid) [3]. Oleic acid is the principal fatty acid in avocado, comprising 45% of its total fatty acids [69], and during the ripening process, palmitic acid content decreases and oleic acid content increases [70]. In terms of its total fat content and fatty acid composition, avocado oil is considered to be similar to olive oil [71]. Other fatty acids present include palmitic and palmitoleic acids with smaller [64] amounts of myristic, stearic, cinolenic, and arachidonic acids [62]. However, the compositions of these fatty acids largely depend on the cultivars, stage of maturity, and part of the fruit and geographic location of plant growth [62]. Avocado spread instead of other fatty alternatives such as butter, cream cheese, and mayonnaise on sandwiches can help significantly reduce the intake of calories, saturated fat, sodium, and cholesterol.

Avocados are notable for their potassium content (>500 mg/100 g of fresh weight), and it provides 60% more than an equal serving of banana [72]. Potassium intake helps to maintain cardiovascular health and muscle function by regulating the blood pressure through the modulation of liquid retention in the body [65]. In addition, potassium regulates the electrolyte balance in the body, which is important for the conduction of electrical signals in the heart (i.e., a steady, healthy heart rate) [65]. The high potassium and low sodium contents in the diet are shown to protect against cardiovascular diseases [3]. Moreover, avocados contain a number of other minerals, including phosphorus, magnesium, calcium, sodium, iron, and zinc (<1 mg/g of fresh weight) [73].

Vitamins such as β-carotene, tocopherol, retinol, ascorbic acid, thiamine, riboflavin, niacin, pyridoxine, and folic acid are also abundantly found in avocado, which are of great importance for overall health and well-being (Table 2) [62,74]. Carotenoids, including lutein, zeaxanthin, and α- and β-carotene found in the pulp of the avocado are potent free radical scavengers [65,74]. The lutein content of avocado is higher than any other fruit, which comprises about 70% of its total carotenoid content [65]. The color of avocado pulp is predominantly attributed to the higher content of xanthophylls (lutein and zeaxanthin). Seasonal variations in the phytochemical profile of avocado especially carotenoids, tocopherol, and fatty acid content have also been reported [65]. Due to their fat-soluble nature, these bioactive compounds have been shown to promote vascular health [65]. Xanthophylls suppress the damage of blood vessels by decreasing the amount of oxidized low-density lipoproteins (LDL) [75]. Additionally, lutein and zeaxanthin have been reported to slow down the progression of age-related macular degeneration, cataracts, and cartilage deterioration [74,76]. Carotenoids in general were demonstrated to protect the skin from ultraviolet radiation-associated oxidation and inflammation [62]. Furthermore, a 68 g serving of Hass avocado contains about 57 mg of phytosterols, which is significantly higher compared to other fruits (about 3 mg per serving) [65]. Avocado phytosterols have been reported to reduce the risks of coronary heart disease [65]. The American Heart Association recommends the consumption of 2–3 g of sterols and stanols per day to promote heart health [65,77]. They are the plant analogues of cholesterol and can be classified into three major groups consisting of β-sitosterol, campesterol, and stigmasterol [78]. The most abundant phytosterol present in avocado is β-sitosterol (76.4 mg/100g), followed by campesterol (5.1 mg/100g) and stigmasterol (<3 mg/100g) [79]. In addition to its cholesterol-lowering activity, β-sitosterol has been demonstrated to inhibit the production of carcinogenic compounds, alleviate symptoms associated with benign prostatic hyperplasia, and strengthen the immune system [79]. In summary, these compounds have been hypothesized to work in conjunction in the prevention of oxidative stress and age-related degenerative diseases [80].

### 1.3. Antioxidant Properties of P. americana

Considering the health risks associated with synthetic antioxidants, the extraction, isolation, and identification of antioxidants from natural sources have become primary research focuses of the food, nutraceutical, and pharmaceutical industries in the recent years [81,82,83]. Annually, over three million tons of avocados are produced worldwide, with only the pulp being used, while the seeds and peel are discarded [2]. Waste utilization by exploiting the phytochemical content of avocado by-products such as seeds and peel will add more value to the avocado industry and may lead to novel product development [84]. Table 3 represents the studies currently available in the literature emphasizing the role of *P. Americana* plant as the source of potent antioxidants. Different parts of the plant, including the leaf, fruit pulp, peel, and seed have been widely studied for their antioxidant properties using conventional spectroscopic assays such as 2,2′-azino-bis (3-ethylbenzothiazoline-6-sulfonic acid diammonium salt (ABTS), 2,2-diphenyl-1-picrylhydrazyl (DPPH), oxygen radical absorbance capacity (ORAC), cupric-reducing antioxidant capacity (CUPRAC), and ferric-reducing ability of plasma (FRAP) as well as more sensitive analytical techniques including high-performance liquid chromatography (HPLC), high-performance liquid chromatography-mass spectrometry (HPLC-MS), gas chromatography-mass spectrometry (GC-MS) and gas chromatography-flame ionization detector (GC-FID). Hass is the most explored avocado variety in terms of its antioxidant properties, which can perhaps be attributed to the popularity and easier availability of this variety. It is evident from the studies performed so far that phenolic compounds (including phenolic and hydroxycinnamic acids, flavonoids, and condensed tannins), carotenoids, α, β, γ, and δ-tocopherols, acetogenins, monounsaturated and polyunsaturated fatty acids are the key antioxidants found in avocado. Moreover, most of these studies have reported significant positive correlations between the phenolic compounds and antioxidant capacity of avocado extracts [84,85,86,87,88]. Phenolic compounds found in avocado were shown to reduce oxidation, inflammation, and platelet aggregation [65]. Several studies have reported that different parts of the avocado plants contain potent phenolic antioxidants such as chlorogenic-, quinic-, succinic-, pantothenic-, abscisic-, ferulic-, gallic-, sinapinic-, *p*-coumaric-, gentisic-, protocatechuic-, 4-hydroxybenzoic-, and benzoic- acids, quercetin, quercetin-3-glucoside, quercetin-3-rhamnoside, vanillin, *p*-coumaroyl-D-glucose, catechins, (−)-epicatechin, and procyanidins (Table 3) [2,28,84,89,90,91,92,93,94,95,96,97]. Among the different parts of avocado investigated in several studies, leaf, peel, and seed extracts have shown consistently greater antioxidant capacity compared to that of the pulp [84,91,94,96,97,98,99,100,101,102,103,104,105,106]. Due to the presence of higher catechin, epicatechin, leucoanthocyanidin, triterpenes, furoic acid, and proanthocyanidin contents, avocado seed extracts have been reported to display greater antioxidant capacity [62,74]. Additionally, the ripening process was also shown to influence the phenolic contents of different parts of the avocado plant [96,107,108]. For example, a study by López-Cobo et al. [96] found a higher content of phenolics in the pulp and seed extracts of overripe avocados compared to their optimally ripe counterparts. It was hypothesized that the increase in the total phenolic content in the overripe fruit was mediated by higher phenylalanine ammonia-lyase activity associated with the ripening process [96]. They also observed an increased concentration of procyanidins in the overripe parts of the avocado, which was probably a result of the hydrolysis of complex tannins after ripening. Avocado peel, seed, and leaf, as the major by-products of the avocado industry, have been demonstrated as rich sources of polyphenolics and antioxidants. More studies developing robust, green, and economical extraction techniques are fundamental to obtain greater yields of potent antioxidants. In vivo and clinical studies to understand the bioavailability of these antioxidants and their potential toxicity are also crucial.

### 1.4. Anticancer Properties of P. americana

**Figure 9:**
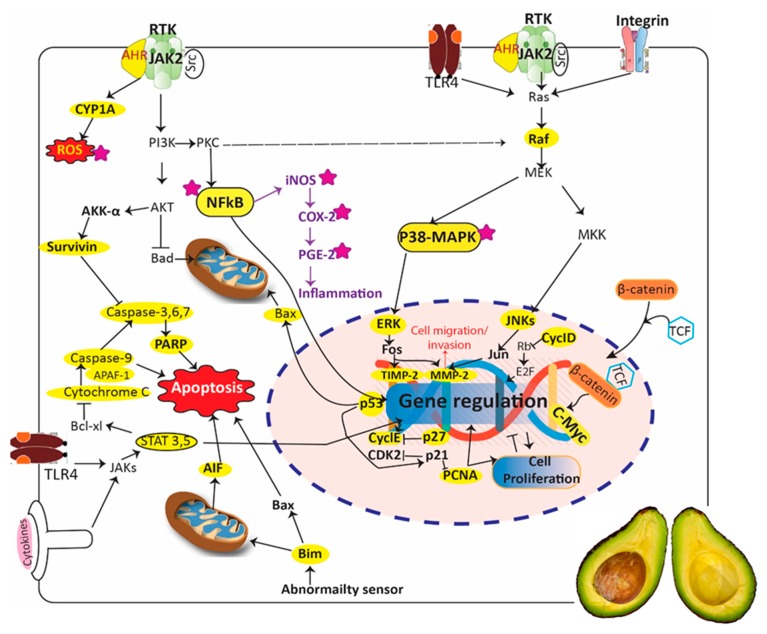
Effect of *Persea americana* (avocado) and its components on different cellular signal transduction pathways. The molecular targets highlighted in yellow play key roles in the proliferation, survival, migration/invasion, and apoptosis of cancer cells. Purple stars indicate the molecular targets involved in inflammatory response.

Cancer causes more deaths than acquired immune deficiency syndrome, tuberculosis, malaria, and diabetes combined [131]. The greatest challenges of anticancer regimens are attributed to the complex mutational landscapes of cancer, late diagnoses, expensive therapeutic options, and the development of resistance to chemo and radiation therapies [132,133]. Chemotherapy-associated side effects and toxicity also make cancer one of the most challenging diseases to treat [133]. Natural products or their derivatives comprised over 45% of the FDA-approved anticancer drugs between 1981–2010 [134]. In the United States, several plant-derived products, either alone or in conjunction with mainstream chemo and radiation therapies are used by approximately 50–60% of cancer patients [132,135]. Therefore, the search for safer alternatives to be used either as mono or adjunct therapy with the standard drugs is becoming a priority in anticancer research [136]. The in vitro cytotoxic properties of avocado against different types of cancer cell lines including breast, colon, liver, lungs, larynx, leukemia, oesophageal, oral, ovary, and prostate have been extensively reported in the literature (Table 4). These properties have also been investigated in preclinical animal models. Interestingly, these in vitro and in vivo studies have not only explored the pulp, the most edible part of the fruit, but also the leaves, peel, and seeds of avocado. Table 4 depicts the major preclinical and clinical studies currently found in the literature emphasizing the potential anticancer activity of avocados. The chemical profiles of different parts of avocado vary among the varieties [84,137]. Therefore, rationally, depending on the chemical profiles, the bioactivities also vary accordingly. Many studies assessing the anti-proliferative activity of avocado did not report the varieties used. However, based on the limited studies that reported the varieties tested, Hass is perhaps the most explored cultivar for its anticancer properties. Molecular mechanistic studies in various cancer cell lines have reported the regulation of different signal transduction pathways, especially the induction of caspase-mediated apoptosis and the involvement of cell cycle arrest by different avocado extracts, their fractions, and isolated compounds (Figure 9, Table 4) [24,26,138,139,140,141,142,143,144,145]. For instance, Dabas et al. [140] recently found out that the methanol extract of Hass avocado seeds induced caspase 3-mediated apoptosis, poly (ADP-ribose) polymerase (PARP) cleavage, and cell cycle arrest at G_0_/G_1_, as well as reduced the nuclear translocation of nuclear factor kappa-B (NF-*κ*B) and downregulated the cyclin D1 and E2 in lymph node carcinoma of the prostate (LNCaP) cells. Parallel observations were made earlier by Lee et al. [144] in MDA-MB-231 (MD Anderson metastasis breast cancer) cells using methanol extracts of avocado seeds and peel. They observed the activation of caspase-3 and its target protein- PARP, in MDA-MB-231 cells. Bonilla-Porras et al. [138] found out that ethanol extracts of avocado endocarp, seeds, whole seeds, and leaves activated transcription factor p53, caspase-3, apoptosis-inducing factor, and oxidative stress-dependent apoptosis via mitochondrial membrane depolarization in Jurkat lymphoblastic leukaemia cells. The acetone extract of avocado pulp rich in lutein, zeaxanthin, β-cryptoxanthin, α-carotene, β-carotene, α-tocopherol, and γ-tocopherol was shown to arrest the PC-3 prostate cancer cells at the G_2_/M phase and increase the expression of p27 protein [24]. The cytotoxic properties of different classes of compounds contribute to the cumulative anticancer activity of avocado. For example, the anticancer effects of the fatty alcohols, carotenoids, and phenolics were further augmented by the potential anticancer effect of norlignans/neolignans (Figure 7) from *P. obovatifolia* [48,49,50,51,52,53].

Scopoletin, a plant coumarin and phytoalexin found in avocado, reduced the carcinogens-induced toxicity and the size of skin papilloma in vivo [26]. Further mechanistic study revealed the modulation of various key cell cycle, apoptotic and tumor invasion markers by scopoletin. Notably, the downregulation of AhR (aryl hydrocarbon receptor), CYP1A1 (cytochrome P450 1A1), PCNA (proliferating cell nuclear antigen), stat-3 (signal transducer and activator of transcription 3), survivin, MMP-2 (matrix metalloproteinase-2), cyclin D1 and c-myc (avian myelocytomatosis virus oncogene cellular homolog); and the upregulation of p53, caspase-3 and TIMP-2 (tissue inhibitor of metalloproteinases-2) by scopoletin were demonstrated [26]. Of note, the expression of p53 and its target genes (~500) regulate a wide range of cellular processes, including apoptosis, cell cycle arrest, and DNA repair [146]. Additionally, the upregulation of TIMP-2 inhibits MMP-2 expression, which consecutively leads to the reduction of cellular migration and invasion (metastasis) [147,148]. Therefore, MMP-2 upregulation has been correlated with poor prognosis and relapse in cancer patients [147]. Another study by Roberts et al. [149] also indicated synergistic interaction between the breast cancer standard drug—tamoxifen—and persin isolated from avocado leaves against MCF-7 (Michigan cancer foundation-7), T-47D, and SK-Br3 breast cancer cells in vitro. The authors reported a significant reduction of tamoxifen IC_50_ values when it was combined with avocado persin. The synergistic interaction was Bim-dependent and mediated by the modulation of ceramide metabolism [149]. Bim is a member of the Bcl-2 (B-cell lymphoma 2) family of proteins that play a key role in the intrinsic (mitochondrial) pathway of apoptosis [150,151]. In particular, Bim is linked with microtubule-stabilizing properties, which mediate the formation of microtubule bundles with subsequent mitotic arrest and apoptosis [139,152].

Chemical synthesis of the most potent anticancer compounds found in avocado has also been carried out in a number of studies [143,145,153,154]. Similar to avocado crude extracts, chemically synthesized avocado peptide PaDef defensin was recently found to induce apoptosis via caspase 7, 8, and 9 expressions in K562 chronic myeloid leukaemia and MCF-7 breast cancer cells in two studies by the same research group [143,153]. Moreover, PaDef defensin was previously demonstrated to have antimicrobial properties [155,156]. The induction of apoptosis and abrogation of the cell cycle were also observed earlier in the human breast, lung, ovarian, and colorectal cancer cells when treated with chemically synthesized avocado β-hydroxy-α,β-unsaturated ketones by Leon et al. [145]. Although many preclinical studies were performed to elucidate the cytotoxicity of extracts derived from different parts of the avocado plant and their components, very few of them have investigated their molecular mechanisms of action. Interestingly, contradicting information regarding avocado extract-induced genotoxicity is also available. For instance, Kulkarn et al. [157] found out that avocado fruit and leaf extracts can induce chromosomal aberrations in human peripheral lymphocytes, with leaf extract being more genotoxic. The same research group later reported that avocado fruit extract can reduce cyclophosphamide-mediated chromosomal aberrations in human lymphocytes [158], which was perhaps due to the antagonistic effects of the extract on cyclophosphamide.

Traditionally, an avocado leaf decoction is used for the treatment of tumors and tumor-related diseases in Nigeria [159]. Despite their health benefits highlighted in numerous reports, clinical studies examining the direct correlation between avocado consumption and the prevention and treatment of cancer are scarce. Only one case-control study involving 243 men with prostate cancer and 273 controls in Jamaica demonstrated that MUFA from avocado may reduce the risk of prostate cancer [160]. However, it should be noted that bioactive compounds that are also commonly found in avocados such as α-carotene, β-cryptoxanthin, lycopene, lutein, and zeaxanthin were found to have inverse associations with cancers of the mouth, larynx, pharynx, and breast in few clinical trials, as highlighted in Table 5 [161,162,163]. According to the USDA, avocados contain a significantly higher amount of glutathione per average serving compared to other fruits [61]. Glutathione is a potent tripeptide antioxidant that plays a major role in detoxification pathways and the reduction of oxidative stress and risk of cancer [62,65]. Notably, it has been linked with the reduction of chemotherapy-associated toxicity and risks of oral cancer in a few clinical studies [57,58,59,164]. Nonetheless, the molecular mechanism of how glutathione reduces the side effects of chemotherapeutic regimens remains largely speculative. In order to precisely understand the anticancer mechanisms of action of avocado extracts and their bioactive compounds, more in vitro and in vivo studies are warranted. As very few studies have identified the solitary bioactive compounds responsible for the growth inhibition of different cancer cells, more research should be undertaken to gain a comprehensive understanding of the chemical profiles of the active extracts. Notably, bioassay-guided fractionation and the subsequent isolation and characterization of biologically active compounds from different parts of the avocado plant may lead to the identification of many novel anticancer compounds. Randomized controlled trials should be designed to evaluate the efficacy of bioactive compounds derived from avocado in the prevention and treatment of different cancer types. Furthermore, the chemoprotective properties of avocado and the possibility of using its bioactive compounds as an adjunct therapy for cancer should also be explored.

### 1.5. Antimicrobial Properties of P. americana

Currently, there is a growing interest in finding alternatives to the synthetic antimicrobial agents that are commonly used in the food and pharmaceutical industries. This is due to the concerns of the consumers regarding the safety of products containing synthetic chemicals and their associated health risks [174]. Seeds (endocarp) and peels (exocarp) being the by-products of the avocado industry are generally disposed of as wastes [175] and have been investigated for their antimicrobial properties. Most of the studies conducted thus far have noted the antimicrobial activity of the extracts derived from different avocado varieties [104,176,177,178], while only a few have reported insignificant antimicrobial activity [101,179]. The antimicrobial activity of avocado extracts might be influenced by (i) the variety of the avocado, (ii) the parts used for investigation (i.e., exocarp, endocarp, or mesocarp), (iii) the solvent type used for extraction, and iv) the bacterial species examined [104,176]. Raymond and Dykes [176] investigated the antimicrobial activity of ethanolic and aqueous extracts of seeds and peels of three different avocado varieties (Table 6). The authors reported that ethanolic extracts had antibacterial activity against both Gram-positive and Gram-negative bacteria (except for *Escherichia coli*) ranging from 104.2 to 416.7 μg/mL, while aqueous extracts exhibited activity against *Listeria monocytogenes* and *Staphylococcus epidermidis*. Rodriguez-Carpena et al. [104] investigated the antibacterial activity of the extracts derived from different avocado parts (peel, seed, and pulp) of a number of varieties against *Bacillus cereus*, *S. aureus*, *L. monocytogenes*, *E. coli*, *Pseudomonas spp*., and *Yarrowia lipolytica*. The highest inhibitory activity against the Gram-positive bacteria- *B. cereus* and *L. monocytogenes* was observed, while *E. coli* was the most sensitive among the tested Gram-negative bacterial species. The authors mentioned that all avocado parts had antimicrobial properties, with pulp (mesocarp) showing the highest activity. In addition, authors reported that the Gram-positive bacteria were more sensitive in comparison to the Gram-negative bacteria [104]. The Gram-negative bacteria have an extra protective outer membrane, which makes them more resistant to antibacterial agents compared to the Gram-positive bacteria [104,180]. β-sitosterol in avocados was also shown to play a key role in strengthening the immune system and the suppression of human immunodeficiency virus and other infections [181]. In particular, it has been found to enhance the proliferation of lymphocytes and natural killer cell activity for invading pathogens [181]. Salinas-Salazar et al. [177] investigated the antimicrobial activity of seed extracts of avocado enriched with acetogenin against *L. monocytogenes* and reported growth inhibition at 37 °C and 4 °C with MIC (minimum inhibitory concentration) values of 15.6 and 7.8 mg/L, respectively. Acetogenins of avocados are fatty acid derivatives with a long unsaturated aliphatic chain (C19–C23) [182,183]. Owing to the structural similarities between acetogenins and fatty acids, authors hypothesized that acetogenins may penetrate the cell membranes of bacteria and physically disrupt their functionality [177]. Indeed, several compounds might be associated in the antimicrobial activity of avocado extracts. Polyphenols have been previously reported for their antimicrobial properties [184]. However, the contribution of the phenolic compounds toward the antimicrobial activity of avocado extracts needs to be investigated. Rodriguez-Carpena et al. [104] found that avocado pulp extract had a higher antimicrobial activity than peel and seed extracts, despite having lower polyphenol content. Future studies should be conducted to isolate individual phenolic compounds from different parts of avocado and investigate their antimicrobial properties.

### 1.6. Anti-Inflammatory Properties of P. americana

Several studies have investigated the anti-inflammatory properties of avocado via modulation of inflammatory responses (Figure 9, Table 7). The aqueous extract of avocado leaves showed an anti-inflammatory effect in vivo by inhibiting carrageenan-induced rat paw oedema [185]. Persenone A, an active constituent of avocado, reduced inducible nitric oxide synthase (iNOS) and cyclooxygenase-2 (COX-2) in murine macrophages [173]. Similarly, (2*R*)-(12*Z*,15*Z*)-2-hydroxy-4-oxoheneicosa-12,15-dien-1-yl acetate, persenone A and B isolated from the avocado fruit, decreased the generation of nitric oxide in mouse macrophages [19]. Avocado oil contains a high amount of oleic acid and essential fatty acids. A study by [186] highlighted the wound-healing properties of avocado fruit oil by increasing collagen synthesis and decreasing inflammation in Wistar rats. They also reported that oleic acid was the predominant unsaturated fatty acid (47.20%) present in the fruit oil [186].

Inflammation in joints causes damage to the joint cartilage due to degenerative changes leading to a loss of joint function and stability [187]. Even though osteoarthritis (OA) is considered a non-inflammatory disease, recent studies have shown that inflammation is a leading cause for the initiation and continuation of the disease process [188]. Non-pharmacological agents that modulate the expression of pro-inflammatory mediators are highly promising as safe and effective ways to treat OA [189]. Avocado–soybean unsaponifiable (ASU) combination represents one of the most commonly used treatments for symptomatic OA [190]. ASU is a combination of avocado oil and soybean oil, which has been accepted as a medication/food supplement in many countries [191]. Three ratios of avocado (A) and soybean (S) unsaponifiable combinations (A:S = 1:2, 2:1, and 1:1) were studied for their anti-inflammatory properties on chondrocyte cells [192]. All the ratios showed significant inhibition compared to the individual extracts on collagenase, stromelysin, interleukin 6 (IL-6), interleukin 8 (IL-8), and prostaglandin E_2_ (PGE2) release. In particular, 1:2 was found to be the most effective combination that exhibited chondroprotective effects in vivo by stimulating glycosaminoglycan and hydroxyproline synthesis and inhibiting the production of hydroxyproline in the granulomatous tissue [192]. In another study, the unsaponifiables of avocado alone indicated a significant chondroprotective effect [193]. Several preclinical and clinical studies conducted in the last few decades have revealed the modulation of different pathways and molecular targets associated with OA pathogenesis by ASU [194]. For instance, the anti-OA properties of ASU are mediated via the suppression of critical regulators of the inflammatory response such as iNOS/COX-2, and PGE-2 [195], and the reduction of catabolic enzymes (matrix metalloproteinases-3 and -13) and [190,196]. Gabay et al. [190] demonstrated the inactivation of the mitogen-activated protein kinases such as the extracellular signal-regulated kinase (ERK 1/2) and NF-*κ*B as the molecular mechanism of action for the anti-inflammatory effects of ASU. A recent study showed the potential bone repair properties of ASU by the modulation of molecular targets *Rankl* and *Il1β*, *RANKL*, and *TRAP* using a rat model [197]. Sterols, the major bioactive components of ASU, have also shown anti-inflammatory activity in articular chondrocytes [198].

A significant reduction of articular cartilage erosion and synovial hemorrhage compared to placebo was observed in horses using ASU extracts [199]. However, the extracts did not reduce signs of pain or lameness in horses. In humans, NSAID (nonsteroidal anti-inflammatory drugs) consumption was reduced in patients with lower limb OA after six weeks of ASU consumption [200]. Furthermore, ASU significantly reduced the progression of joint space loss in patients with hip OA [201]. Another study by Maheu et al. [202] demonstrated slow radiographic progression in hip OA using ASU treatment. They also reported that the treatment was well tolerated by patients, even though the clinical outcome did not change. Interestingly, a recent study showed that the intake of ASU extract decreased the pain symptoms and an improved the quality of life in patients with OA of the temporomandibular joint [203].

Other studies have combined ASU with bioactive compounds such as epigallocatechin gallate (EGCG), and α-lipoic acid (LA) [189,204,205]. Interestingly, contrary to previous research, Heinecke et al. [204] reported a slight inhibition of COX-2 expression and PGE_2_ production in activated chondrocytes. However, when ASU was combined with EGCG, both mediators were more significantly inhibited than their mono treatments [204]. Another study by Ownby et al. demonstrated that this combination inhibited the gene expression of interleukin-1 beta (IL-1β), tumor necrosis factor- α (TNF-α), IL-6, COX-2, and IL-8 in activated chondrocytes [189]. The combination of ASU with LA showed a more significant suppression of PGE_2_ production in activated chondrocytes than ASU or LA alone [205].

The implementation of ASU in the treatment of other inflammatory diseases has also been explored. In particular, ASU has shown efficacy against periodontal disease by modulating the expression of transforming growth factor beta 1 (TGF-β1), TGF-β2, and bone morphogenetic protein 2 (BMP-2) [206]. Additionally, a recent study demonstrated that ASU can repair periodontal disease within seven days [207]. These results underline the significant anti-inflammatory properties of avocado mediated via multiple signal transduction pathways and their role in the potential treatment of various inflammatory diseases.

### 1.7. Effect of P. americana on Cardiovascular Health and Diabetes

Clinical have shown a positive effect on cardiovascular health and lipid profiles with the presence of avocado in the diet [65,208]. It has been observed that the intake of avocado in a balanced diet had a great impact on preventing cardiovascular diseases as a result of the low cholesterol levels. Grant in 1959 [209] conducted the first avocado clinical trial where 0.5 to 1.5 avocados were incorporated in the diet of 16 male patients, and showed a significant decrease or the same total serum cholesterol level with no increase in weight. In particular, avocado phytosterols were found to inhibit cholesterol absorption and synthesis by mimicking its molecular structure, which resulted in lowered total cholesterol levels in the body [210]. A randomized, controlled trial was conducted on 45 obese patients where the patients were categorized into three major groups—(i) moderate-fat diet, (ii) low-fat diet, and (iii) moderate-fat diet with the incorporation of one avocado (AV). A major decrease in the total cholesterol levels in all the groups was observed from the baseline, while the AV group had a greater reduction in LDL-C and non-high-density lipoprotein (non-HDL) [67]. According to the results from the National Health and Nutrition Examination Survey (NHANES), people who consumed avocados had an improved diet quality due to increased vegetable intake and reduced sugar consumption [211]. Therefore, vascular damage and heart diseases can be reduced to a great extent by the inclusion of avocados in a diet [211]. In another study, the inclusion of avocado in a meal increased satisfaction with a decrease in actual eating in obese adults, which indirectly had a positive effect on the body mass index (BMI) and reduced the chances of cardiovascular diseases [212]. An increase in the satisfaction (by 23%), decrease in eating (by 28%), and blood insulin were observed in comparison to the control group [212]. A systematic review was conducted by Silva Caldas et al. [213] to study the effects of avocado on the cardiovascular health of adults, and after including eight articles from the initial 234 studies, they concluded that the presence of MUFA, specifically oleic fatty acid in avocado has been linked with its cardioprotective effects.

A study done by Carvajal-Zarrabal et al. [214] found out that avocado oil had a significant contribution toward the metabolic syndrome, as it reduced the inflammatory events and exhibited positive results in the biochemical indicators when they administered avocado oil in 25 rats divided into various groups such as a control group, a basic diet group with 30% sucrose, and a basic diet plus olive oil and avocado oil. Extensive biochemical markers were studied, and the presence of avocado oil seemed to have reduced the triglycerides and LDL levels, which reduced the cardiovascular risks [214]. Cohort studies performed recently on the BMI of individuals after the intake of avocados showed a considerable reduction in weight gain compared to the control, which consecutively lowered various cardiovascular problems associated with obesity [215]. Another report in 2018 [216] analyzed studies on the intake of avocado and cardiovascular risks from MEDLINE, Cochrane Central, and the Commonwealth Agricultural Bureau, and found a significant increase in HDL cholesterol concentration with heterogeneity associated with avocado intake. However, no significant reduction in LDL cholesterol and serum total cholesterol was mentioned in this report [216].

The indigestible carbohydrates abundantly found in avocado are reported to prevent diabetes and regulate blood cholesterol [217]. The glycemic index can be defined as a comparative ranking of carbohydrate in foods according to their effect on blood sugar levels [218]. Despite its carbohydrate content, the glycemic index rating of avocado is quite low. In rats, various aqueous concentrations of *P. americana* seed extract exhibited hypoglycemic and antihyperglycemic effects by significantly decreasing the blood glucose levels [219], highlighting its potential in the management of diabetes mellitus. Another study conducted to investigate the effect of avocado paste on rats with a hypercholesterolemic diet with high fructose showed lower levels of blood sugar and significant reduction of fat accumulation in the liver, which was attributed to the presence of bioactive compounds (polyphenols, fiber, and carotenoids) [220]. Investigation on the inhibitory effects of phenolic extract from the avocado pulp, leaves, and seed on various type 2 diabetes enzymes (α-amylase and α-glucosidase) was also performed [221]. The peel extract exhibited the highest inhibition against α-amylase and α-glucosidase, while the leaf extract significantly inhibited the α-glucosidase. In a recent study, the glycemic and lipoprotein profiles of the obese middle-aged adult were improved when the carbohydrate was replaced with avocados in a meal [222]. The participants were divided into three different groups: control group (0 g), half avocado (half-A, 68 g), and whole avocado (whole-A, 136 g). In comparison to the control group, the half-A and whole-A group showed decreased glycemic and insulinemic response over 6 h [222].

### 1.8. Bioavailability and Pharmacokinetic of Compounds from P. americana

Avocado is a relatively unique fruit, containing high levels of water and fat-soluble vitamins, plant sterols, MUFA, and phytochemicals [223]. Avocado has been shown to improve the absorption of nutrients when used in combination with other foods and supplements [224]; however, research on the pharmacokinetics of the avocado components alone is limited.

Vitamin A is fat-soluble in nature and present in many foods as retinol and in its provitamin A form (carotenes). In particular, liver, fish, and cheese are rich sources of vitamin A. Carotenes (provitamin A) are converted to vitamin A in the body. However, plant-based foods typically present a challenging matrix for the utilization of vitamin A, hindering the absorption and conversion of provitamin A to vitamin A [225]. Many commonly consumed plant-based foods contain higher levels of provitamin A such as sweet potato (709 µg/100 g), carrots (835 µg/100 g), and spinach (469 µg/100 g), especially compared to avocado (7 µg/100 g) [223]. Nevertheless, the levels of vitamins in the food are trivial if not absorbed and converted to their active chemical forms for the body to utilize. The absorption of provitamin A from plant sources is typically poor. In an in vitro digestion model, the accessibility of β-carotene in raw carrots was 1–3% and lycopene was <1% [226]. The consumption of lipid-rich food has been shown to improve the absorption of fat-soluble vitamins, including vitamin A [227]. The presence of soluble fats during digestion facilitates the formations of mixed micelles, which facilitate absorption [228].

The absorptions of provitamin A including β-carotene, α-carotene, β-cryptoxanthin, lutein, and zeaxanthin were enhanced when co-consumed with avocado. This can perhaps be attributed to the high MUFA content of avocado. In salsa, the absorption of lycopene and β-carotene was increased by 4.4 and 2.6 times respectively when avocado was added. In salad (150 g), the addition of avocado (24 g) increased the absorption of α and β-carotene and lutein by 7.2, 15.3, and 5.1 times, respectively [224]. In addition to the improved absorption, avocados were shown to enhance the utilization of provitamin A by increasing the conversion rate to vitamin A in participants with low conversion efficacy [229]. The enhanced absorption of provitamin A has been attributed to the improved formation of mixed micelles in the lumen, increasing solubility and facilitating uptake by enterocytes. Improved vitamin A uptake has been observed with other high lipid foods such as eggs and oil [230]. Likewise, the consumption of salad rich in carotenes, with canola oil, resulted in significantly higher carotene concentrations in chylomicrons [231]. As avocado is a rich source of fat and high in monosaturated fatty acids, it presents an alternative from sources high in unsaturated fats.

Avocado is the most concentrated source of β-sitosterol in commonly consumed Western fruits [79]. Plant sterols share similar chemical structures with cholesterol; however, they are poorly absorbed compared to cholesterol, (with about 10% systematically absorbed compared to 50–60% for cholesterol) [232]. Similar to other lipophilic compounds, phytosterols are incorporated into mixed micelles before being taken up by enterocytes [233]. Plant steroids may assist in lowering cholesterol absorption by acting as a competitive inhibitor. Interestingly, plant sterols have also been observed to lower dietary carotene plasma levels by 10–20% [234]. As avocados have been reported to increase carotene absorption and subsequent plasma levels, this effect may be overcome by the benefit of the other lipid components present. Due to its unique fruit matrix high in plant sterols and MUFA, avocados may provide an enhanced absorption of lipophilic compounds compared to other fruits and vegetables. As established for vitamin A and carotene, it is likely that the absorption of other lipophilic compounds may similarly be enhanced by consumption with avocado. Within avocado, this may apply to vitamin E, vitamin K, chlorophylls, and phytochemicals such as acetogenins. Further pharmacokinetic research is necessary to determine if the absorption of other lipophilic compounds is enhanced in combination with avocado. The current literature does not provide any information regarding the effect of avocado matrix on the absorption of water-soluble vitamins and phytochemicals. Moreover, further pharmacokinetic research should be directed to understand the bioavailability of pharmaceutically promising phytochemicals such as acetogenins from avocado.

## 2. Conclusions and Future Direction

Several preclinical studies performed in the last few decades lay emphasis on the unique nutritional and phytochemical composition of avocado and their potential in the treatment and prevention of different diseases. Some studies have underlined its importance as the source of lead molecules for drug discovery due to the abundance of novel chemical skeletons. The cumulative effects of avocado components in the prevention and treatment of oxidative stress and age-related degenerative diseases are also indicated in a few studies. However, more comprehensive in vitro, in vivo, and clinical investigations are fundamental to significantly expand the understanding of the molecular mechanisms of action of its phytochemicals for developing subsequent therapeutic and nutritional interventions against cancer, diabetes, inflammatory, microbial, and cardiovascular diseases. Interestingly, despite its popularity as a “superfood”, clinical studies evaluating the therapeutic potential of avocado for the prevention and management of different ailments are limited in the literature. More investigations to understand the bioavailability and pharmacokinetics of avocado phytochemicals and antioxidants are also crucial to determine their clinical efficacy and potential toxicity. Regardless of the recent food trends and marketing gimmicks of “superfoods”, variety is fundamental for a balanced healthy diet. As many studies have revealed the complex synergistic interactions among different phytochemicals present in food matrices, studies to understand the possible synergy between bioactive compounds from avocado and other fruit and vegetables will help formulate diet-based preventive strategies for many diseases. A few reports have indicated the role of avocado in improving the bioavailability of nutrients from other plant-based foods. Therefore, consuming avocados with other fruit and vegetables as a part of the diet can be beneficial to human health.

## Figures and Tables

**Table 1:** Metabolites isolated from *Persea* species.

Compound Name and Synonyms	Source	Extracts of Different Parts Used	Biological Significance	Reference
**Fatty alcohols**				
(2R,4R)-1,2,4-trihydroxyheptadec-16-yne [Avocadyne] 1,2,4-trihydroxyheptadec-16-ene 2,4-methylene-dioxyheptadec-16-ene-1-ol 1-acetoxy-2,4-dihydroxyheptadec-16-yne (2R,4R)1,2,4-Nonadecanetriol. (2R,4R,6E)-6-Nonadecene-1,2,4-triol (2R,4R,16E)-16-Nonadecene-1,2,4-triol [Avocadenol D]	*P. americana*	Pulp and seeds	Inhibition of the dengue virus replication. Cytotoxic, insecticidal, antimycobacterial, and trypanocidal activity.	[10,11,12,13,21]
(Z,Z)-1-Acetoxy-2-hydroxy-4-oxo-heneicosa-12,15-triene (Z,Z,E)-1-Acetoxy-2-hydroxy-4-oxo-heneicosa-5,12,15-triene 1,2,4-trihydroxyheptadec-16-ene	*P. americana*	Idioblast cells of pulp	Antifungal activity	[14]
(2R,4R)16-Heptadecene-1,2,4-triol and the following derivatives: 1,2, or 4 acetate (1,2), (1,4) or (2,4) di acetate 1-hexadecanolyl derivative (Avocadoin)	*P. americana*	Peel, idioblast cell, and leaves	Antifungal, cytotoxic, and insecticidal activity.	[11,14,15]
2-(isopropyl)-(2E,4E)-16-Heptadecene-1,2,4-triol 2-(isopropyl), 1,4-di-acetyl-(2E,4E)-16-Heptadecene-1,2,4-triol	*P. gratissima*	Leaves	-	[7]
(2E,5E,12Z,15Z) 1-Hydroxy-2,5,12,15-heneicosatetraen-4-one 1-Hydroxy-2,12,15-heneicosatrien-4-one	*P. americana*	-	-	[7]
Acetyl-2-nonanol	*P. gratissima*	Leaves	-	[7]
Persin Tetrahydropersin Isopersin Tetrahydropersin	*P. americana*	Idioblast oil cells	Surfactant and emulsifier, nutrient, membrane stabilizer, energy source, and energy storage.	[8,16,17]
1-Acetoxy-2-hydroxy-16-heptadecen-4-one	*P. americana*	Pulp		[18]
Persenone A and B	*P. americana*	Pulp	Nitric oxide and superoxide generation inhibitors.	[19]
Secosubamolide	*P. americana*	Bark	Cytotoxic activity	[20]
***Phenolics***				
Gallic acid 3,4-Dihydroxyphenylacetic acid 4-Hydroxybenzoic acid Vanillic acid p-Coumaric acid Ferulic acid Quercetin	*P. americana*	Pulp oil and varied by ripening and peeling	Antioxidant activity	[28]
(+)-Catechin (−)-Epicatechin Neochlorogenic acid procyanidins	*P. americana*	By-products	Antioxidant and neuroprotective activity.	[22]
Proanthocyanidins B1, B2 and A-type trimer	*P. americana*	Seeds	Cytotoxic to HaCat cells.	[23]
Tocopherols (Vitamin E) α-tocopherol γ-tocopherol	*P. americana*	Pulp and pulp oil varied by ripening and peeling	Antioxidant activity	[24,28]
(*E*)-Chlorogenic acid (Caffeylquinic acid, Caffetannic acid, Helianthic acid, Igasuric acid)	*P. americana*	*-*	Antioxidant, antimicrobial (antibacterial and antiviral) hepatoprotective, cardioprotective, anti-hypertension, anti-obesity, anti-inflammatory, antipyretic, neuroprotective, central nervous system stimulator.	[7,25]
Scopoletin	*P. americana*	*-*	Anti-oncogenic and antioxidant activity.	[7,26]
4-Hydroxycinnamoylputrescine (4-Coumaroylputresine)	*P. gratissima*	*-*	Nutrient, promotes cell multiplication of tobacco explants.	[7,27]
**Carotenoids**				
Lutein zeaxanthin β-cryptoxanthin α-carotene β-carotene (pro-vitamin A, retinol)	*P. americana*	Pulp and pulp oil varied by ripening and peeling	Cytotoxic to prostate cancer cell lines, antioxidant, reduces the photosensitivity reactions in erythropoietic protoporphyria patients.	[24,28]
10’,11’-Didehydro-5,8,11’,12’-tetrahydro-10’-apo-β-carotene-3,5,8-triol 5,8-Epoxy-5,8-dihydro-10’-apo-β,ψ-carotene-3,10’-diol	*P. americana*	Pulp	Surfactant and emulsifier, nutrient, membrane stabilizer, energy source and energy storage.	[8,29]
α-Citraurin (3-Hydroxy-8’-apo-ε-caroten-8’-al)	*P. americana*	Pulp		[30]
***Carbohydrates***				
Perseulose	*P. gratissima*	Leaves, fruit, and seeds	Nutrient, membrane stabilizer, energy source and energy storage.	[44]
d-erythro-l-galacto-Nonulose	*P. americana*	Pulp		[45]
d-erythro-l-gluco-Nonulose	*P. americana*	Pulp		[46]
d-erythro-d-galacto-Octitol	*P. gratissima*	Pulp		[47]
d-manno-2-Heptulose	*P. gratissima* *P. americana*	Pulp		[7,47]
d-glycero-d-manno-2-Octulose	*P. gratissima*	Pulp		[47]
**Furan derivatives**				
Avocadofuran B (2-Heptadecylfuran) *P. americana*		Pulp	Insecticidal activity	[31,32]
Avocadofuran A (2-Pentadecylfuran)	*P. americana*	Idioblast oil cells		
Avocadienofuran	*P. americana* *P. indica*	Seed oil pulp	-	[33,34]
Perseafuran [*(E)-*2-(1-Pentadecenyl) furan]				
Isoavocadienofuran		Seeds		
Avocadenofuran	*P. americana*	Pulp		[18]
Avocadynofuran	*P. americana* and *P. indica*	Pulp		[18,33]
**Furanone derivatives**				
Obtusilactone A (Borbonol)	*P. americana, P. borbonia* and other *Persea spp.*	Idioblast oil cells	Antifungal and anticancer activity.	[35,36]
Isoobtusilactone A (Borbonol 2)	*Persea spp*	Idioblast cell oil of pulp	Antifungal and anticancer activity.	[35,37]
Majorynolide	*P. major*	*-*	Cytotoxic, weak antimycobacterial activity.	[33]
16,17-Dihydro-Majorynolide	*P. major* and *P. indica*	*-*		
**Diterpenoids**				
Perseanol Vignaticol Indicol	*P. indica*	Branches	Insecticidal and antifeedant activity.	[39,40]
Ryanodol 2,3-DidehydrocinnzeylanoneAnhydrocinnzeylanoneGarajonone			Insecticidal and toxic to mice.	[41,42,43]
**Norlignans/Neolignans/Lignans**				
Perseal A ((7’R,8’S)4’,7’-Dihydroxy-3,3’-dimethoxy-8,9-dinor-4,8’-oxylignan-7-al) Perseal B ((7’S,8’S) 4’,7’-Dihydroxy-3,3’-dimethoxy-8,9-dinor-4,8’-oxylignan-7-al) Obovatinal Perseal C Perseal D Perseal E ((7’S,8’S) 4,7’-Epoxy-3’,4’-dihydroxy-5,5’-dimethoxy-8,9-dinor-3,8’-lignan-7-al) ObovatenObovatifol	*P. obovatifolia*	Branches	Cytotoxic activity	[48,49,50,51,52]
Lingueresinol	*P. lingue*	Bark	-	[53]
**Miscellaneous**				
(6S,7E,9Z) Abscisic acid-13-Hydroxy, 13-O-β-D-glucopyranoside	*P. americana*	Seeds	Derivative of abscisic acid (plant hormone involved in seed and bud dormancy).	[7]
Dimethyl sciadinonate	*P. americana*	-	Growth inhibitor of silkworm larvae.	[7,54]
(3β,5α,24R) Stigmast-7-en-3-ol; (Schottenol, 22-Dihydrochondrillasterol, 22,23-Dihydro-α-spinasterol, Poriferast-7-en-3-ol)	*P. americana*	Pulp oil	Protective role by cholesterol metabolism modulation (liver x receptor agonist).	[55]
Perseapicroside A	*P. mexicana*	-	-	[56]
Glutathione	*P. americana*	*-*	Anticancer and antioxidant activity.	[57,58,59]
12-Tridecenal	*P. bombycina*	Essential oil	-	[60]

**Table 2:** Pulp composition of *Persea americana* (avocado) [61].

Nutritional Composition	Unit	Value Per 100 g	1 Fruit 136 g	1 Serving 30 g
**1. Proximate**				
Water	g	72.3	98.4	21.7
Energy	kcal	167	227	50
Energy (insoluble fiber adjusted)	kcal	148	201	44
Protein	g	1.96	2.67	0.59
Total lipid (fat)	g	15.41	21	4.62
Ash	g	1.66	2.26	0.5
Carbohydrate	g	8.64	11.8	2.59
Fiber	g	6.8	9.2	2
Sugars	g	0.3	0.41	0.09
Starch	g	0.11	0.15	0.03
**2. Minerals**				
Calcium	mg	13	18	4
Iron	mg	0.61	0.83	0.18
Magnesium	mg	29	39	9
Phosphorus	mg	54	73	16
Potassium	mg	507	690	152
Sodium	mg	8	11	2
Zinc	mg	0.68	0.92	0.2
Copper	mg	0.17	0.23	0.05
Manganese	mg	0.15	0.2	0.05
Selenium	ug	0.4	0.5	0.1
**3. Vitamins and Phytochemicals**				
Vitamin C	mg	8.8	12	2.6
Thiamine	mg	0.08	0.1	0.02
Riboflavin	mg	0.14	0.19	0.04
Niacin	mg	1.91	2.6	0.57
Pantothenic acid	mg	1.46	2	0.44
Vitamin B-6	mg	0.29	0.39	0.09
Folate, dietary folate equivalents	μg	89	121	27
Choline total	mg	14.2	19.3	4.3
Betaine	mg	0.7	1	0.2
Vitamin B-12	μg	0	0	0
Vitamin A	μg	7	10	2
β-Carotene	μg	63	86	19
α-Carotene	μg	24	33	7
β-Cryptoxanthin	μg	27	37	8
Lutein + zeaxanthin	μg	271	369	81
Vitamin E (α-tocopherol)	mg	1.97	2.68	0.59
Tocopherol β	mg	0.04	0.05	0.01
Tocopherol γ	mg	0.32	0.44	0.1
Tocopherol δ	mg	0.02	0.03	0.01
Vitamin K1 (phylloquinone)	μg	21	28.6	6.3
**4. Lipids**				
Fatty acids, total monounsaturated	g	9.799	13.3	2.94
16:1	g	0.698		
17:1	g	0.01		
18:1	g	9.066		
20:1	g	0.025		
Fatty acids, total saturated	g	2.126	2.9	0.64
8:0	g	0.001		
16:0	g	2.075		
18:0	g	0.049		
Fatty acids, total polyunsaturated	g	1.816	2.47	0.55
18:2	g	1.674		
18:3	g	0.125		
18:3 n-3 c,c,c (ALA)	g	0.111		
18:3 n-6 c,c,c	g	0.015		
20:3	g	0.016		
Cholesterol	mg	0	0	0
Stigmasterol	mg	2	3	1
Campesterol	mg	5	7	2
β-sitosterol	mg	76	103	23

**Table 3:** Antioxidant properties of *Persea Americana* (avocado).

Variety	Part Studied	Types of Extract	Detection Assays	Major Findings	Type of Antioxidants	References
Hass	Pulp and peel + pulp	Expeller pressed oils	ABTS and HPLC-PDA	Higher antioxidant capacity, α-tocopherol and β-carotene content were observed in oils from the unpeeled microwave-dried pulp of ripe and unripe avocado.	Oils from the pulp of ripe unpeeled microwave-dried avocado had significantly greater phenolic acid and quercetin contents.	[28]
Hass	Peel	50% (*v/v*) ethanol using accelerated solvent extraction	HPLC coupled to ultra-high-definition accurate-mass-QTOF	Sixty-one compounds belonging to 11 families were identified.	Procyanidins, flavonols, hydroxybenzoic, and hydroxycinnamic acids.	[90]
Hass	Seeds and seed coat	Accelerated solvent extraction	DPPH, TEAC, ORAC, HPLC-DAD-ESI-QTOF-MS	Significant antioxidant activity was observed in both seed and seed coat extracts. A total of 84 compounds were identified, among which 45 were phenolic compounds.	Condensed tannins, phenolic acids, and flavonoids.	[91]
Hass	Pulp	Oil extracted with or without ultrasound	HPLC	Similar quantities of α, β, γ, and δ-tocopherols and phenolic compounds were detected both with and without ultrasound extractions.	Tocopherols and phenols.	[109]
Hass	Seeds	Methanol and 50% (*v/v*) ethanol	HPLC, ABTS, FRAP, ORAC and methoxy radical scavenging activity by EPR	50% (*v/v*) ethanol extract displayed greater antioxidant capacity in the ORAC, FRAP, and ABTS assays.	Chlorogenic acid, (−)-epicatechin, catechins and procyanidins.	[2]
Hass	Peel and seeds	Aqueous extract	ORAC	Peel extract showed higher antioxidant capacity than seed extract.	Epicatechin and chlorogenic acid were found in both extracts.	[101]
Hass	Pulp, peel, and seeds	Hexane to eliminate lipids and 80% methanol for phenolic extraction	HPLC-DAD-ESI-QTOF-MS	Higher concentrations of phenolic compounds were detected in the pulp and seed extract of overripe than in pulp and seed of optimally ripe fruit. The concentration of procyanidins increased after ripening.	Nine compounds in pulp, three in peel and three in seed. Procyanidins to degree of polymerization 2 to 6, and 13 were identified and quantified.	[96]
Hass	Peel, pulp, and seeds	Ultrasonic extraction with 80% (*v/v*) ethanol	DPPH, and ABTS	Seed and peel extracts exhibited greater antioxidant values and phenolic content than the pulp extract.	-	[102]
Hass	Peel, pulp, and seeds	Different solvents for different assays	DPPH and spectroscopic	All extracts exhibited significant antioxidant capacity. The seed extract had the greatest antioxidant activity, total phenolic content, and flavonoids compared to that of peel and pulp.	Carotenoids, phenolic compounds, flavonoids, vitamin c and tocopheryl acetate were detected in all extracts.	[106]
Hass	Pulp	Aqueous and ethanolic	FRAP and DPPH	Harvesting seasons affected the antioxidant capacity.	Positive correlations between FRAP and total phenolics, DPPH and total phenolics	[85]
Hass	Pulp	Hydrophilic and lipophilic extracts	DPPH, TEAC and ORAC	Higher antioxidant capacity values were obtained from lipophilic extracts compared to hydrophilic extracts.	A positive correlation was observed between DPPH/TEAC assays with palmitoleic, oleic, linoleic, α-linolenic acids.	[108]
Hass	Pulp	Acetone with 2,6-ditert-butyl-4-methylphenol, sodium carbonate, and sodium sulfate	HPLC-PDA	Seasonal variations in carotenoid were observed and α-tocopherol was detected.	Carotenoid such as: All-trans-neoxanthin; all-trans-violaxanthin; all-transneochrome; 9-cis-neoxanthin; all-trans-lutein-5,6-epoxide; chrysanthemaxanthin; lutein; zeaxanthin; β-cryptoxanthin; α-carotene; β-carotene were identified along with α-tocopherol.	[110]
Hass	Pulp	Tetrahydrofuran	DPPH	Low antioxidant activity.	A slight positive correlation against stearic acid content.	[111]
Hass	Leaves, pulp, peel, and seeds	Freeze-dried samples	FRAP, 4-dinitrophenylhydrazine and HPLC	The leaf, peel, and seed extracts had greater antioxidant capacity than that the pulp extracts. C7 sugars such as mannoheptulose and perseitol contributed to the antioxidant capacity of the pulp.	Vitamin C, anthocyanin, and C7 sugars.	[100]
Hass and Fuerte	Peel and seeds	80% (*v/v*) ethanol with ultrasonic extraction	ABTS, DPPH, FRAP, and HPLC-ABTS	Peel extracts of both varieties displayed higher antioxidant capacity in the ABTS and FRAP assays compared to their seed extracts, whereas in the DPPH assay, seed extracts showed greater antioxidant activity.	Peel: procyanidin B2 and epicatechin Seed: trans-5-*O*-caffeoyl-D-quinic acid, procyanidin B1, catechin, and epicatechin.	[97]
Hass and Fuerte	Pulp, peel, and seeds	Ethyl acetate, 70% (*v/v*) acetone, and 70% (*v/v*) methanol	CUPRAC, DPPH, and ABTS	Acetone (70% *v/v*) was found to be the most effective solvent for extracting antioxidants. Peel and seed extracts exhibited greater antioxidant values in all three assays compared to pulp.	Peels and seeds: catechins, procyanidins, and hydroxycinnamic acids Pulp: hydroxybenzoic and hydroxycinnamic acids and procyanidin.	[104]
Hass and Shepard	Seeds and peel	80% (*v/v*) methanol	HPLC-PAD, HPLC-ESI-MS, DPPH, ABTS and ORAC	The peel extracts displayed a higher total phenolic compound content and antioxidant activity in comparison to the seed extracts. Hass variety had a higher antioxidant capacity, which might be attributed to its procyanidin dimers and catechins than the Shepard variety.	Seed and peel extracts contained flavanol monomers, proanthocyanidins, and hydroxycinnamic acids. In addition, flavonol glycosides were detected in seed extracts.	[94]
Hass, Lamb-Hass, and Rugoro	Pulp	Methanol, ethanol, acetone, and ethyl acetate	HPLC-DAD-ESI-TOF	Seventeen compounds were identified using standards. Twenty-five compounds were tentatively identified.	Quinic acid, succinic acid, pantothenic acid, *p*-coumaroyl-D-glucose, abscisic acid, pentadecylfuran, avocado furan, and oleic acid were the most common compounds among the three avocado varieties.	[92]
Hass, Quintal, Margarida, and Fortuna	Peel, pulp, and seeds	Ethanol	ABTS, DPPH, FRAP	Peel extract of the Quintal variety showed the highest antioxidant capacity in all three assays. A similar trend was observed in terms of total phenolic and flavonoid contents.	Phenolics and flavonoids might contribute to the antioxidant capacity.	[99]
Hass, Bacon, Fuerte, Pinkerton, Rincon, and Orotawa	Pulp	Methanol	UHPLC-HE-MS	Pulp extracts had 19 individual phenolic compounds. A decrease in concentration of epicatechin concentration was observed with fruit ripening.	Gallic acid, sinapinic acid, vanillin, *p*-coumaric acid, gentisic acid, protocatechuic acid, 4-hydroxybenzoic acid, chlorogenic acid, and benzoic acid.	[89]
Hass, Hass Motril, ColinV 33, Gem, Harvest, Jiménez 1, Jiménez 2, Lamb Hass, Marvel, Nobel, Pinkerton, Sir Prize and Tacambaro	Pulp	Methanol	GC coupled to APCI-TOF MS and FID	Twenty-seven compounds were quantified by GC-APCI-MS. Seven compounds are quantified by GC-FID. The concentration of organic acids, flavonoids, and vitamins decreased, whereas phenolic acids, ferulic acids, or *p*-coumaric acids increased with the ripening process.	Quinic, ferulic, chlorogenic and *p*-coumaric acids, epicatechin, and quercetin.	[93]
Booth 7	Pulp	Sodium acetate	ABTS	Total antioxidant capacity gradually increased with the ripening process. Treatment with aqueous 1-methylcyclopropene (1-MCP) significantly delayed the accumulation of total soluble phenolics, flavonoids, and total antioxidant capacity.	-	[112]
Collinson	Pulp	80% methanol and acetone	ABTS, DPPH, and FRAP	Lipophilic extracts displayed greater antioxidant capacity in the ABTS and DPPH assays compared to hydrophilic extracts. The opposite trend was observed in the FRAP assay.	-	[113]
Fortuna	Fresh and dried seeds	Water, 70% (*v/v*) ethanol, 70% (*v/v*) methanol, and partition with n-hexane chloroform, ethyl acetate, and n-butanol	Spectroscopic and HPLC	Ethanol extract of dried seed showed 50, 38, and 24 mg/g of dry matter of total phenol, condensed tannins, and flavonoid contents, respectively. HPLC study revealed epicatechin (4.7 μg/mL), rutin (2.8 μg/mL), and chlorogenic acid (1.4 μg/mL) and quercetin in the extract.	Epicatechin, rutin, chlorogenic acid, quercetin.	[114]
Fortuna	Pulp	Oil extracted with SCO_2_ and compressed LPG	DPPH	The SCO_2_-extracted oil displayed higher antioxidant activity in the range of 17.4–82.5% compared to LPG-compressed oil.	-	[115]
Fortuna	Pulp	Lyophilized and cold pressed oil	GC-FID and GC-MS	A greater concentration of α-tocopherol and squalene were achieved with cold pressing.	α-tocopherol and squalene.	[116]
Fuerte	Pulp	Different solvents	FRAP, SOD and HPLC	Increase in the total antioxidant activity, SOD activity, and α-tocopherol content was observed in the presence of 1-MCP and low O_2_.	-	[117]
Lula	Pulp	Oil extracted with water at high temperatures	HPLC and spectroscopic assays	Greater quantity of α-tocopherol was detected compared to β, γ, and δ-tocopherols. In addition, sterols and carotenoids were also reported.	Tocopherols, sterols, and carotenoids were potent antioxidants.	[118]
Mexican landrace	Peel	Methanol	DPPH	Antioxidant values in the range of 53.31–307.33 mmol trolox equivalents/fresh weight were reported.	Activity can be attributed to anthocyanins.	[119]
Slimcado, Booth 7, Booth 8, Choquette, Loretta, Simmonds, and Tonnage	Pulp, peel, and seeds	Acetone, water, acetic acid	HPLC-MS, ORAC and DPPH	Seed extracts exerted the highest antioxidant activity, phenolic content, and procyanidins followed by peel and pulp. Significant correlations were observed among antioxidant capacities, phenolic contents, and procyanidins. Antioxidant activity can be attributed to the procyanidin content.	Catechin, epicatechin, A- and B-type dimers, A- and B-type trimers, tetramers, pentamers and hexamers were identified in peels and seeds.	[84]
-	Pulp	Supercritical CO_2_/ ethanol extracts	HPLC	Supercritical CO_2_ + ethanol at 200 bar and at 40 °C and 60 °C yielded significantly higher α-tocopherol content.	α-tocopherol	[120]
-	Seeds and pulp	Lipid	ABTS and DPPH	Seed extracts exhibited significantly greater antioxidant activity in both assays. Dose-dependent antioxidant activity was observed for both extracts.	-	[98]
-	Pulp	Oil extracted with mechanical pressing	DPPH	Greater antioxidant values were observed when the avocado pulp was dried at 60 °C under ventilation, and mechanical pressing was used for the oil extraction compared to vacuum oven and Soxhlet extraction.	α-tocopherol, phenolic compounds, carotenoids.	[121]
-	Seeds	Ultrasonic extraction with water	ORAC	Total antioxidant capacity increased with an increase in ultrasonic power. Positive correlation was observed between total polyphenolic content and antioxidant capacity.	-	[86]
-	Pulp	Acetone and its fractions	ORAC, HPLC-PDA/MS-TOF	Fractions with lipophilic acetogenins exhibited the highest antioxidant capacity.	1-acetoxy-2,4-dihydroxy-n-heptadeca-16-ene; Persediene; Persenone-C; Persenone-A; Persenone-B; Persin, and 1-acetoxy-2,4-dihydroxy-heneicosa-12,15-diene.	[122]
-	Leaves	50% ethanol extract	Spectroscopic, LC–ESI-MS, LCMS-IT-TOF	Glycosylated flavonoids were detected.	Quercetin-3-glucoside and quercetin-3-rhamnoside.	[95]
-	Seeds	Different concentrations of ethanol	ORAC	The antioxidant values increased with temperature. However, it was negatively impacted by ethanol concentration.	-	[123]
-	Leaves, pulp, peel, and seeds	1M HCL and methanol	DPPH and FRAP	Greater DPPH radical scavenging activity, total phenol and flavonoid content were observed in leaf extracts. The peel extract showed the greatest FRAP value.	-	[103]
-	Pulp and seeds	50% (*v/v*) ethanol	DPPH and FRAP	Seeds extracts showed significantly greater antioxidant values compared to that of pulp in both assays. Similar trend was observed for total phenolic content.	-	[105]
-	Peel	Different concentrations of ethanol	DPPH	Maximum antioxidant activity when extraction was performed with 48% (*v/v*) ethanol under agitation for 20 min at 70 °C and solvent-to-solid ratio (*v/w*) 20.	Positive correlation was observed between total phenolic content and antioxidants.	[88]
-	Seeds	Different concentrations of ethanol	DPPH	Extraction for 60 min with 30% (*v/v*) ethanol at 70 °C with a solvent to-solid material ratio of 8 yielded the maximum antioxidant capacity.	Positive correlation was observed between total phenolic content and antioxidants.	[87]
-	Leaves	Methanol, ethanol, cold and hot water	DPPH, FRAP, and hydroxyl radical scavenging ability	Significant antioxidant activity was observed in all three assays.	Antioxidant activity might be contributed by the phenolics and flavonoids.	[124]
-	Pulp	Oils extracted using Soxhlet, subcritical CO_2_ (SCO_2_) and ultrasound	ABTS, FRAP, and β-carotene bleaching	SCO_2_-extracted oil displayed significantly greater (*p* < 0.05) antioxidant capacity in all three assays compared to Soxhlet or ultrasound-extracted oils.	Strong positive correlations (*p* < 0.01) were found between α and γ tocopherols and antioxidant activity.	[125]
-	Leaves	Powdered leaves	Spectroscopic	Vitamin C, tannins, alkaloids and phenolic content were reported.	-	[126]
-	Pulp	Lipid-soluble bioactive	DPPH, reducing power, metal chelating, nitric oxide scavenging, hydrogen peroxide scavenging, hemoglobin-induced linoleic acid system	Exhibited lower antioxidant properties compared to vitamin C.	-	[127]
-	Pulp	Methanol + water	ABTS and TBARS	Lower antioxidant activity was reported compared to other fruits tested in the study.	-	[128]
-	Leaves and seeds	Water	DPPH, NO radical scavenging activity, inhibition of degradation of deoxyribose, Fe (II) chelating ability	Higher phenolic content and radical scavenging activity were observed in leaf extract. However, it showed lower iron chelation activity compared to the seed extract.	-	[129]
-	Seeds	Different solvents and fractions	DPPH	One fraction exhibited a radical scavenging activity of 81.6%.	-	[130]

ABTS: 2,2′-azino-bis (3-ethylbenzothiazoline-6-sulfonic acid) diammonium salt. TEAC: Trolox equivalent antioxidant capacity. DPPH: 2,2-Diphenyl-1-picrylhydrazyl. ORAC: Oxygen radical absorbance capacity. HPLC-PDA: High-performance liquid chromatography–photodiode array. HPLC-DAD-ESI-QTOF-MS: High-performance liquid chromatography–diode array detector–electrospray ionization–quadrupole time-of-flight mass spectrometry. HPLC-ESI-QTOF-MS: High-performance liquid chromatography–electrospray ionization–quadrupole time-of-flight mass spectrometry. FRAP: Ferric reducing ability of plasma. CUPRAC: Cupric reducing antioxidant capacity. SOD: Superoxide dismutase. HPLC-MS: High-performance liquid chromatography mass spectrometry. HPLC-ESI-MS: High-performance liquid chromatography–electrospray ionization–mass spectrometry. LC–ESI-MS: Liquid chromatography–electrospray ionization–mass spectrometry. UHPLC-HE-MS: Ultra high-performance liquid chromatography–heated electrospray–mass spectrometry. TBARS: Thiobarbituric acid reactive substances. HPLC-DAD-ESI-TOF: High performance liquid chromatography–diode array detector–electrospray ionization–time of flight. GC-APCI-TOF-MS: Gas chromatography–atmospheric pressure chemical ionization–time-of-flight mass spectrometry. GC-APCI-TOF-FID: Gas chromatography–atmospheric pressure chemical ionization–time-of-flight–flame ionization detector.

**Table 4:** Preclinical and clinical studies highlighting the anticancer properties of *Persea americana* (avocado).

Preclinical Studies						
Variety	Parts	Type of Extracts	Bioactive Compounds	Type of Cell Lines	Major Findings and Molecular Mechanisms of Action	References
Hass	Seeds	Methanol	-	MCF-7 breast, H1299 lung, HT29 colon, and LNCaP prostate cancer cells	Dose-dependent inhibition of all cells with IC_50_ values 19–132 µg/mL after 48 h of treatment. In LNCaP prostate cancer cells, the induction of caspase 3-mediated apoptosis, PARP cleavage, downregulation of cyclin D1 and E2, cell cycle arrest at G_0_/G_1_ phase and reduction of nuclear translocation of nuclear factor kappa B (NF-*κ*B) were observed.	[140]
Hass	Seeds	High-speed countercurrent chromatographic fraction of methanol-water partition (M7)	Proanthocyanidins B1, B2 and A-type trimer. Traces of abscisic acid glucosides.	HaCaT immortalized nontumorigenic human epidermal cells	Significant inhibition of cell proliferation, increased LDH activity. Molecular mechanisms of action were not investigated.	[23]
Hass	Pulp	Chloroform-soluble	Two aliphatic acetogenins- (2S,4S)-2,4-dihydroxyheptadec 16-enyl acetate] and 2 [(2S,4S)-2,4-dihydroxyheptadec-16-ynyl acetate.	83–01-82CA human oral cancer cell line, MEK overexpressing cell line 83–01-82CA/MEKCA	The two aliphatic acetogenins targeted the EGFR/RAS/RAF/MEK/ERK1/2 cancer pathway by synergistically inhibiting c-RAF (Ser338) and ERK1/2 (Thr202/Tyr204) phosphorylation.	[165]
Hass	Pulp	Chloroform	-	83-01-82CA human oral cancer and TE1177 normal epithelial cell lines	In the oral cancer cells, the extract induced apoptosis by increasing the levels of reactive oxygen species by twofold to threefold. Apoptosis was not induced in the normal cell line.	[141,142]
Hass	Pulp	Acetone	Lutein, zeaxanthin, β-cryptoxanthin, α-carotene, and β-carotene, α-tocopherol and γ-tocopherol.	LNCaP androgen-dependent and PC-3 androgen-independent prostate cancer cell lines	Inhibited the growth of both the prostate cancer cell lines. Arrested PC-3 cells at the G_2_/M phase and increased the expression of p27 protein.	[24]
Lulu	Unripe fruit pulp	95% (*v/v*) ethanol extracts and its fractions	1,2,4-Trihydroxynonadecane, 1,2,4-Trihydroxyheptadec-16-ene and 1,2,4-Trihydroxyheptadec-16-yne.	A-549 human lung, MCF-7 human breast, HT-29 human colon, A-498 human Kidney, MIA PaCa-2 human pancreatic carcinoma*,* PC-3 human prostate cancer cells	All three compounds were active against six human tumor cell lines and exhibited selectivity against PC-3 cells. Molecular mechanisms were not studied.	[21]
-	Seeds	Ethanol extract and its hexane and dichloromethane fractions	-	Lung A549 and gastric BGC823 cancer cells	Growth inhibition at 200 μg/mL. The IC_50_ values and molecular mechanisms of action were not investigated.	[166]
-	Pulp and seed extracts	Lipids	Fatty acids, hydrocarbon, and sterols.	HCT116 colon and HePG2 liver cancer cell lines	Seed extract showed greater activity against HCT116 (IC_50_ < 4 µg/mL) and HePG2 (IC_50_ < 20 µg/mL) cell lines compared to the pulp extract. Molecular mechanisms of action were not investigated.	[98]
-	Seeds	Chloroform extracts and its soluble methanol fraction (FML) and non-soluble methanol fraction (FTML).	-	MCF-7 breast cancer cell line	Chloroform extract, FML, and FTML inhibited cell growth in a dose-dependent manner and displayed IC_50_ values of 94.87, 34.52, and 66.03 µg/mL, respectively. FML induced apoptosis and arrested cells at the subG_1_/G_0_ phase.	[167]
-	Leaves	Silver nanoparticles		MCF-7 breast and HeLa cervical cancer cells	Dose-dependent cytotoxicity was observed at concentrations above 50 μM in MCF-7 but not in HeLa cells. Downregulation of p53 expression was observed in both cell lines.	[168]
-	Leaves	Aqueous-ethanol (5% v/v)	-	Larynx cancer tissue	Significant increase in adenosine deaminase activity in cancerous tissues derived from 13 patients who underwent surgery for larynx cancer (median age of 57 years) compared to noncancerous (*r* = 0.60, *p* = 0.029) tissues.	[169]
-	Seeds	Fraction of ethanol extract	Triterpenoid	MCF-7 breast and HepG2 liver cancer cells	Inhibited MCF-7 (IC_50_ = 62 µg/mL) and HepG2 (IC_50_ = 12 µg/mL) cells with no activity against normal cells. Molecular mechanisms of action were not investigated.	[170]
-	Pulp	Ethanol, chloroform, ethyl acetate, and petroleum.	-	Esophageal squamous cell carcinoma and colon adenocarcinoma cell line	Moderate activity. The IC_50_ values and molecular mechanisms of action were not investigated.	[171]
-	Pulp	Aqueous	-	A549 lung, HepG-2 liver, HT-29 colon, and MCF-7 breast cancer cells.	Exhibited LC_50_ values in the range of 13.3–54.5 µg/mL against the tested cell lines. Molecular mechanisms of action were not investigated.	[172]
-	Root bark	Methanol extract and its fractions.	4-hydroxy-5-methylene-3-undecyclidenedihydrofuran-2 (3H)- one	MCF-7 breast cancer cell line	Antiproliferative activity with an IC_50_ value of 20.48 μg/mL with induction of apoptosis.	[36]
-	Endocarp, whole seed, seed and leaves	Ethanol	-	Jurkat lymphoblastic leukemia cells	Induced significant oxidative stress-dependent apoptosis via mitochondrial membrane depolarization. Activated transcription factor p53, protease caspase-3, and apoptosis-inducing factor (APAF).	[138]
-	Pulp	50% (*v/v*) Methanol	-	Human lymphocyte cells	Chemoprotective against cyclophosphamide-induced chromosomal aberrations at 200 mg/kg body weight.	[158]
-	Seeds and peel	Methanol	-	MDA-MB-231 breast cancer cells	Apoptosis due to activation of caspase-3 and its target protein, PARP.	[144]
-	Leaves	-	Persin	In vitro: MDA-MB-231, MCF-7, and T-47D breast cancer cells In vivo: Quackenbush lactating mice	In vitro: Persin selectively arrested cells at the G_2_/M phase and induced caspase-dependent apoptosis. Apoptosis was dependent on the expression of Bim protein, which also indicated the microtubule-stabilizing properties of persin. Overall, MCF-7 and T-47D cells were more sensitive to persin compared to MDA-MB-231. In vivo: Persin exerted cytotoxicity in the lactating mammary epithelium.	[139]
				MCF-7, T-47D, and SK-Br3 breast cancer and MCF-10A human mammary epithelial cells.	Synergistic interaction between tamoxifen and persin against the tested breast cancer cells was observed. Significant reduction of IC_50_ values of tamoxifen when combined with 13.8 μmol/L of persin. The synergistic cytotoxicity was Bim-dependent and mediated by the modulation of ceramide metabolism.	[149]
-	Fruit	-	Persenone A	In vitro: RAW 264.7 mouse macrophage cells In vivo: Female ICR mice (7 weeks old)	Downregulated the expression of iNOS/COX-2 (nitric oxide synthase/cyclooxygenase-2) in macrophage cells. When applied topically, reduced the generation of H_2_O_2_ in mouse skin.	[173]
-	Fruit	-	(2R)-(12Z,15Z)-2-hydroxy-4-oxoheneicosa-12,15-dien-1-yl acetate (1), persenone A (2) and B (3)	HL-60 acute promyelocytic leukemia and RAW 264.7 mouse macrophage cells.	Suppressed the growth of HL-60 cells (compound 1, IC_50_ = 33.7; compound 2, IC_50_ = 1.4; compound 33, IC_50_ = 1.8 μM). Inhibited nitric oxide generation induced by lipopolysaccharide in combination with interferon-γ in RAW 264.7 cells.	[19]
-	-	-	Scopoletin	In vivo: Skin papilloma in mice induced by 7,12-dimethylbenz(a)anthracene and croton oil	Reduced carcinogen-induced toxicity and led to decrease in the size of skin papilloma. Downregulated AhR, CYP1A1, PCNA, stat-3, survivin, MMP-2, cyclin D1, and c-myc, and upregulated p53, caspase-3, and TIMP-2.	[26]
**Chemical synthesis**			**Type of cell lines**		**Major findings and molecular mechanisms of action**	**References**
Antimicrobial peptide-PaDef defensin			K562 chronic myeloid leukemia cells		Cytotoxic with an IC_50_ value of 97.3 μg/mL. Activated caspase-8 and induced the expression of TNF-α.	[153]
			MCF-7 breast cancer cell line		Inhibited the growth in a concentration-dependent manner (IC_50_ = 141.62 µg/mL). Induced cytochrome c, APAF-1, and the caspase 7 and 9 expressions, loss of mitochondrial Δψm and enhanced the phosphorylation of MAPK p38.	[143]
Persin and tetrahydropersin			Breast cancer: MCF-7, T-47D, MDA-MB-468, MDA-MB-157, SkBr3, Hs578T, MDA-MB-231 cells, normal mammary epithelial MCF-10A cells, Ovarian cancer: OVCAR3 and IGROV-1 cells Prostate cancer: PC-3 and LNCaP cells		Persin was more potent compared to tetrahydropersin against most of the tested cancer cell lines with IC_50_ values in the range 15.1 ± 1.3 to more than 39 μM. Molecular mechanisms of action was not studied.	[154]
β-Hydroxy-α,β-unsaturated ketones			A2780 human ovarian, SW1573 lung, HBL-100 human breast, T-47D human breast and WiDr colorectal cancer cells.		GI_50_ values in the range of 0.5–3.9 μM. Induced apoptosis and dose-dependent cell cycle arrest in the S and G_2_/M phase.	[145]
**Case-control studies**						
**Type of cancer**			**Major findings**			**References**
Prostate cancer			A study involving 243 men with prostate cancer and 273 controls in Jamaica reported that monounsaturated fat from avocado was associated with reduced risk of prostate cancer.			[160]

**Table 5:** Clinical studies demonstrating the anticancer activity of bioactive compounds that are also commonly found in *Persea americana* (avocado).

Bioactive Compounds	Type of Cancer	Type of Study	Major Findings	References
Carotenoids- α-carotene, β-cryptoxanthin, lycopene, and lutein/zeaxanthin	Breast cancer	A nested case-control study in women consisting of 604 breast cancer cases and 626 controls.	In women with high mammographic density, plasma levels of carotenoids reduced breast cancer risk significantly (40–50% reduction, *p* < 0.05).	[162]
		An ancillary study involving 207 women ages 18 to 70 years who had been successfully treated for early-stage breast cancer.	An inverse association between total plasma carotenoid concentrations and the oxidative stress biomarkers (urinary 8-hydroxy-2′-deoxyguanosine and 8-isoprostaglandin-F2α) was observed.	[163]
	Larynx, pharynx and oral cancers	The study population involving 52 patients curatively treated for early-stage larynx, pharynx or oral cavity during 1997–2001.	An inverse association was observed between individual/grouped xanthophylls and urinary F2-isoprostanes (F_2_-IsoPs), a biomarker of oxidative stress. However, individual/grouped carotenes did not show such association with F_2_-IsoPs.	[161]
Glutathione	Advanced colorectal carcinoma	A randomized, double blind, placebo-controlled trial in 52 patients.	Prevented of oxaliplatin-induced neuropathy without reducing the clinical efficacy of oxaliplatin.	[57]
	Ovarian cancer	A multicenter, randomized, double-blind, parallel group design with 51 women.	Reduced the cisplatin-associated toxicity and improved the quality of life.	[58]
	Oral cancer	A population-based case-control study involving 1,830 Caucasian participants (855 cases and 975 controls) in during 1984–1985 in the United States.	Reduced oral cancer risk was associated with glutathione when fruit and vegetable were commonly consumed raw.	[59]

**Table 6:** Summary of studies that have been conducted that investigated the antimicrobial activity of *Persea americana* (avocado).

Variety/ies	Bacteria	Highlights	Reference
Hass Shepard Fuerte	*Listeria monocytogenes* *Staphylococcus epidermidis* *Staphylococcus aureus* *Enterococcus faecalis* *Escherichia coli* *Salmonella Enteritidis* *Citrobacter freundii* *Pseudomonas aeruginosa* *Salmonella Typhimurium* *Enterobacter aerogenes*	The antimicrobial activity of peel and seed extracts was evaluated. Ethanol extracts showed antimicrobial activity against both Gram-positive and Gram-negative bacteria (except *E. coli*). Aqueous extracts had antimicrobial activity against *L. monocytogenes* and *S. epidermidis*.	[176]
Hass Fuerte	*Bacillus cereus* *S. aureus* *L. monocytogenes* *E. coli* *Pseudomonas spp.* *Yarrowia lipolytica*	All avocado parts had antimicrobial activities. Pulp showed the highest antimicrobial activity. Gram-positive bacteria were found to be more sensitive than Gram-negative bacteria.	[104]
Hass	*L. monocytogenes*	The antilisterial properties of an enriched acetogenin extract from avocado seed were determined. Seeds had higher acetogenin content than pulp. The antimicrobial effect was probably caused by increased membrane permeability.	[177]
Lorena Hass	*S. aureus* *E. coli*	Extracts did not have antimicrobial activity against *S. aureus* ATCC 29213 and *E. coli* ATCC 25922	[179]
Hass	*Listeria innocua* *E. coli* *Lactobacillus sakei* *Weissella viridescens Leuconostoc mesenteroides*	Peel and seed extracts did not present antimicrobial activity against any bacteria analyzed.	[101]

**Table 7:** Anti-inflammatory properties of *Persea americana* (avocado) extracts, compounds, and combinations.

Extracts and Compounds	Key Findings and Molecular Mechanism of Action	Reference
Leaf aqueous extract	Reduced carrageenan-induced rat paw oedema.	[185]
Persenone A	Reduced inducible nitric oxide synthase (iNOS) and cyclooxygenase-2 (COX-2) in activated murine macrophages.	[173]
Avacado oil	Promoted increased collagen synthesis and decreased inflammation in wound healing on incisional and excisional cutaneous wound models in Wistar rats.	[186]
(2*R*)-(12*Z*,15*Z*)-2-hydroxy-4-oxoheneicosa-12,15-dien-1-yl acetate, persenone A and B	Decreased nitric oxide generation in activated mouse macrophages.	[19]
Avocado–Soybean Unsaponifiables (ASU)	Inhibited collagenase, stromelysin, IL-6, IL-8, and prostaglandin E_2_ (PGE2) release in activated human articular chondrocytes.	[192]
	Stimulated glycosaminoglycan and hydroxyproline synthesis, and inhibited the production of hydroxyproline in the granulomatous tissue of mice model.	[193]
	Suppressed critical regulators of the inflammatory response such as PGE-2 and COX-2 in activated human chondrocytes.	[195]
	Decreased catabolic enzymes, matrix metalloproteinases-3 and -13 expressions via inactivating the expression of MAPKs (ERK 1/2) and nuclear factor kappa-B (NF-*κ*B) in activated mouse or human chondrocytes.	[190]
	Reduced pro-inflammatory cytokines such as TNF-α, IL-1β, and iNOS expression in activated chondrocytes and THP-1 monocyte and macrophages.	[196]
	Exhibited a promising result on the bone repair by modulating the molecular targets of *Rankl* and *Il1β*, *RANKL*, *TRAP* in rat model.	[197]
	Decreased pain symptoms in patients with osteoarthritis of the temporomandibular joint.	[203]
	Modulated the expression of TGF-β1, TGF-β2, and BMP-2 in activated human periodontal ligament and human alveolar bone cells.	[206]
ASU + Epigallocatechin gallate	Inhibited COX-2 expression and PGE_2_ production in activated equine chondrocytes.	[204]
	Inhibited the gene expression of IL-1β, TNF-α, IL-6, COX-2, and IL-8 in activated equine chondrocytes.	[189]
